# Comprehensive metabolomics-based analysis of sugar composition and content in berries of 18 grape varieties

**DOI:** 10.3389/fpls.2023.1200071

**Published:** 2023-06-09

**Authors:** Haixia Zhong, Vivek Yadav, Zhang Wen, Xiaoming Zhou, Min Wang, Shouan Han, Mingqi Pan, Chuan Zhang, Fuchun Zhang, Xinyu Wu

**Affiliations:** The State Key Laboratory of Genetic Improvement and Germplasm Innovation of Crop Resistance in Arid Desert Regions (Preparation), Key Laboratory of Genome Research and Genetic Improvement of Xinjiang Characteristic Fruits and Vegetables, Institute of Horticultural Crops, Xinjiang Academy of Agricultural Sciences, Urumqi, China

**Keywords:** sugar content, grape varieties, berry morphology, GC-MS, physicochemical characteristics

## Abstract

Xinjiang is the largest grape-producing region in China and the main grape cultivation area in the world. The Eurasian grape resources grown in Xinjiang are very rich in diversity. The sugar composition and content are the main factors that determine the quality of berries. However, there are currently no systematic reports on the types and contents of sugars in grapes grown in Xinjiang region. In this research, we evaluated the appearance and fruit maturity indicators of 18 grape varieties during fruit ripening and determined their sugar content using GC-MS. All cultivars primarily contained glucose, D-fructose, and sucrose. The glucose content in varieties varied from 42.13% to 46.80% of the total sugar, whereas the fructose and sucrose contents varied from 42.68% to 50.95% and 6.17% to 12.69%, respectively. The content of trace sugar identified in grape varieties varied from 0.6 to 2.3 mg/g. The comprehensive assessment by principal component analysis revealed strong positive correlations between some sugar components. A comprehensive study on the content and types of sugar will provide the foundation to determine the quality of grape cultivars and effective ways to utilize resources to improve sugar content through breeding.

## 1 Introduction

Sugar composition and content are the main factors used to measure the quality of fruit, and they are essential for superior enological characteristics (Torregrosa et al., 2015; Kanayama, 2017). Sugar content in grape berries is another major factor that determines fruit quality (Zhang et al., 2022a). Sugar accumulation plays an important role in the synthesis of flavor substances and secondary metabolites and is a key indicator to judge whether the fruit is fully mature (Brumos, 2021). The growth and development of berries can be divided into three stages. In the first stage, the accumulation of sugary substances is less, the content of acidic substances is higher, and the fruit grows faster (Bigard et al., 2019; Durán-Soria et al., 2020). Compared to the first and third stages of development, the growth and development of berries are much faster in the second stage. In the third stage, the fruit growth is slower, and the accumulation of various compounds, including sugar substances and anthocyanins, takes place (Manning et al., 2001; Tadeo et al., 2020). The content of acidic substances begins to decline, and the fruit gradually becomes soft (Agasse et al., 2009; Lecourieux et al., 2014). The accumulation of sugar substances in grape berries is dominated by the accumulation of glucose and fructose, supplemented by the accumulation of sucrose (Fontes et al., 2011). These three sugar components and contents play a key role in the formation of fruit quality (Varandas et al., 2004; Liu et al., 2017). Glucose and fructose in grapes account for 97.88%–99.86%, and the proportion of both in the ripening stage is 0.74–1.05 (Li et al., 2020). Sucrose is the main form of sugar transportation and is decomposed into glucose and fructose at maturity. The glucose content in the green fruit stage is higher than that in the overripe stage, and the accumulation of sucrose accounts for less than 4% of the total sugar (Liu et al., 2006). The sugar accumulation in grapes is in direct proportion to their growth and development (Manning et al., 2001). Therefore, the accumulation of fructose, glucose, and sucrose is an important carbohydrate substance affecting fruit quality. The movement and transformation of products of photosynthesis are linked to the sugar accumulation in fruit, and the activity of enzymes involved in sugar metabolism is key to controlling the source-sink relationship (Ruan, 2014; Zhu et al., 2018; Parker et al., 2020).

The accumulation of carbohydrates in fruits is closely related to the activity of metabolism-related enzymes (Liu et al., 2006; Li et al., 2017). High acid invertase (AI) activity in grape berries was observed during growth, development, and maturation, and the activities of sucrose synthase (SS) and sucrose phosphate synthase (SPS) are directly related to the content of sugars (Bilska-Kos et al., 2020; Liao et al., 2022). SS is mainly located in the cytoplasm and can control the functionality of the fruit sink organs. SS catalyzes the following reversible reactions: fructose+UDPG ←→ sucrose+UDP (guanosine diphosphate) (Durán-Soria et al., 2020). SS-c showed high activity in the early stage of grapefruit development and decreased activity at maturity (Jiang et al., 2014; Liu et al., 2022b). The study found that sucrose synthase exhibits strong activity in the decomposition direction during the early stage of peach fruit development, while sucrose synthase shows strong activity in the synthesis direction during the late stage (Vimolmangkang et al., 2016; Zhang et al., 2019). With the exception of weak SS-c activity during fruit setting and harvest, SS-s activity in orange fruit is strong during other periods (Shi et al., 2016). In watermelon, the activity of SS-c is consistently stronger than that of SS-s, indicating that sucrose synthase is mainly involved in catalyzing the reversible cleavage of sucrose into fructose (Liu et al., 2006). Neutral invertase (NI) and aldose reductase (AI) are negatively correlated with sucrose content and positively correlated with hexose content (Wang et al., 2021). Previous findings have shown that sucrose accumulation in citrus fruits during the young fruit and expansion stages increases gradually with decreasing invertase (INV) activity (Gutiérrez-Miceli et al., 2002). Additionally, it has been observed that acid invertase activity is strong during the young fruit stage of *Myrica rubra* and consistently high in the ‘Feizixiao’ fruit of litchi (Huicong et al., 2002). In tomato, invertase activity is low during the early stages of development but gradually increases as the development process accelerates, reaching its highest point during maturity. However, sucrose content is low during the later stages (Quinet et al., 2019; Durán-Soria et al., 2020). Therefore, there is a growing interest in exploring various sugar types in fruit and developing an understanding of sugar accumulation based on variety differences.

The grapevine (*Vitis vinifera* L.) is cultivated worldwide due to its lucrative nature as a fruit crop that thrives in various climates (Grassi and De Lorenzis, 2021). Grapes have a high economic value because, in addition to being eaten fresh, they are used to produce juice and wine. Additionally, grapes contain numerous healthy nutrients beneficial to human health. In recent decades, grape cultivation has significantly expanded in scale throughout China (Zhong et al., 2022). Xinjiang, owing to its unique location, long hours of sunshine, and wide range of natural resources, has become the most important region in China for producing high-quality berries (Zhang et al., 2022a). The total cultivated land area for grapevines in Xinjiang has reached 26,000 hectares (Zhang et al., 2022a; Zhang et al., 2022b). Recent studies showed that this grape growing region is an important center from a domestication and evolution point of view. Many elite cultivars have been reported with specific berry characteristics. Comparative studies are essential in determining fruit quality and exploring the potential of crops in the region. Furthermore, different types of sugar metabolism-related enzymes contribute differently to sugar accumulation, but there is a correlation between them. Fruit sugar accumulation is achieved through the cooperation of sugar metabolism-related enzymes. Although various scientific studies and reports have revealed the composition and content of sugar units in popular grape varieties, there have been no systematic comparative studies on the types and contents of sugar composition between popular and local elite grape varieties. Therefore, it is of great significance to study the variation in individual and total soluble sugar contents among different grape varieties. In this paper, 18 grape varieties from Xinjiang were used as research materials. Their appearance and internal basic indicators during fruit ripening were analyzed, and their sugar content was detected and analyzed by GC-MS. By clarifying the content and types of sugar in Xinjiang grape germplasm resources, conducting a comprehensive evaluation, and identifying grape varieties rich in sugar, it will be possible to regulate sugar, which is an important quality, and find more effective ways to improve sugar content, ultimately achieving the breeding goal.

## 2 Materials and methods

### 2.1 Grape verities and field cultivation

The experiment was conducted at the grape research base of the Horticulture Institute (87.28’E, 45.56’N), Xinjiang Academy of Agriculture Sciences, under the fruit quality control post of the national grape industry technology system, from July to December 2018. The experimental base covers an area of four hectares and is equipped for irrigation, fertilization, weeding, and spraying. The base is well constructed and developed. In the current study, 18 varieties of fresh grapes were used as test materials over the course of 6 years. These varieties covered the main types with varying maturities, shapes, and colors, as outlined in **Table 1**. All varieties were planted on the same location in 2012 and began bearing fruit in 2014. Grapes plants were spaced in 1×3.5 m, and standard cultivation practices were followed. For each variety, 15 healthy trees with uniform growth and the same flowering period were selected. Each group consisted of five trees with three biological repeats and berries were harvested at their optimum technological maturity as per the OIV resolution VITI 1/2008 (OIV, 2008).

**Table 1:** Basic profile of 18 varieties used in the current study.

Name of Cultivar	Abbreviation	Species	Maturity	Colour	Seeds
Summer Black	SB	*V.vinifera* × *V.Labrusca*	Early-maturing	Black	Seedless
Bronx seedless	BS	*V.vinifera* × *V.Labrusca*	Early-maturing	Light red	Seedless
Crimson seedless	CS	*V.vinifera*	Extremely late maturing	Red	Seedless
Flame seedless	FS	*V.vinifera*	Early-maturing	Red	Seedless
Rizamat	RZ	*V.vinifera*	Medium	Red	Seedy
XinYu	XY	*V.vinifera*	Medium	Red	Seedless
Wuhecuibao	WHCB	*V.vinifera*	Early-maturing	Green	Seedless
Shine Muscat	SM	*V.vinifera* × *V.Labrusca*	Medium	Green	Seedy
Victoria	VT	*V.vinifera*	Early-maturing	Green	Seedy
Black Monukka	MK	*V.vinifera*	Mid early maturity	Purple	Seedless
Red Globe	RG	*V.vinifera*	Late maturing	Red	Seedy
Thompson seedless	TS	*V.vinifera*	Mid early maturity	Green	Seedless
Centennial seedless	CT	*V.vinifera*	Medium	Green	Seedless
Munake	MN	*V.vinifera*	Late maturing	Green	Seedy
Yatomi Rosa	FHYDM	*V.vinifera*	Mid early maturity	Red	Seedy
Huozhouheiyu	HZ	*V.vinifera*	Mid early maturity	Black	Seedless
Huozhouhongyu	HZHY	*V.vinifera*	Mid early maturity	Red	Seedless
Melissa	ML	*V.vinifera*	Late maturing	Green	Seedless

### 2.2 Fruit sample collection, handling, and extraction

After the fruit sample was cleaned well, the exocarp and mesocarp were quickly separated with a scalpel, cut into small pieces, and put in liquid nitrogen. Some extra samples were transferred to the laboratory and stored in a freezer at -80°C. The grape berry samples were freeze-dried in a vacuum, and the freeze-dried berry was ground (30 Hz, 1.5 minutes) to powder by using a grinder with zirconia beads. Weigh 20 mg grape powder and add 500 μL methanol: isopropanol: water (3:3:2 V/V/V) extract, vortex for 3 min, and ultrasonic in ice water for 30 min. Centrifuge, add an internal standard, and then freeze dry. The chemical derivation method is as follows: a small molecule carbohydrate sample and 100 μL methoxy ammonium salt pyridine (15 mg/mL) solution were mixed, and the mixture was incubated at 37°C for 2 h. Then, 100 mL of BSTFA solution was added to the mixture and incubated at 37°C for 30 min to obtain the derivatized solution. The solution was diluted with n-hexane to an appropriate concentration and stored in a brown sampling bottle for analysis.

### 2.3 Determination of basic indicators in the field

The vertical and horizontal diameters of fruit were measured with an electronic digital vernier caliper, and the fruit shape index was calculated (Barbagallo et al., 2020). The weight of a single fruit was measured with a 1/10000 electronic analytical balance. The content of total soluble solids concentration (°Brix) was determined by a PAL-1 digital refractometer, and the total acid was determined by a PAL-BX/ACID-2 Brix acid-meter.

### 2.4 GC-MS analysis

Sugar determination was carried out using the GC-MS method (Medeiros and Simoneit, 2007; Ruiz-Matute et al., 2011; Milkovska-Stamenova et al., 2015; Wang et al., 2022). Briefly, sugar substances in grape berries were analyzed by gas chromatography (Agilent 7890B), mass spectrometry (7000 d), and a DB-5MS column. With helium as the carrier gas, the flow rate is 1 mL/min. The injector and source temperatures were maintained as per standard procedures. The oven temperature ramp progress was maintained at 170°C, 250°C, 280°Agilent 7890B), mass spectrometry (7000 d), and a DB-5MS column. With helium as the carrier gas, the flow rate is 1 mL/min. The injector and source temperatures were maintained as per standard procedures. The oven temperature ramp progress was maintained at 170°C, 250°C, 280°C, and 310°C. The details of the GC-MS analysis for specific parameters are presented in **Table 2**.

**Table 2:** Mass spectrometer condition and specific parameters during GC-MS analysis.

Mass spectrometry conditions	Parameter
Sample quantity	3 μL
Front Inlet Mode	3:1
Carrier Gas	Helium
Column	DB-5MS (30 m x 0.25 mm x 0.25 μm)
Column Flow	1mL min^−1^
Oven Temperature Ramp	170°C (1min), raised to 250°C at a rate of 10°C/min, raised to 280°C at a rate of 4°C /min, raised to 310°C at a rate of 25°C/ min, 310°C (3.72min)
Front Injection Temperature	250°C
Transfer Line Temperature	240°C
Ion Source Temperature	230°C
Quad Temperature	150°C
Electron Energy	70eV
Scan mode	SIM
Solvent Delay	3.5min

Based on the GC-MS platform, MetWare software (Wuhan, China, http://www.metware.cn/) was used to do a qualitative and quantitative analysis of the sugar components (Wang et al., 2022). For each group of samples, three biological replications were maintained. Sugar standards were procured from Olchemim, Aladdin (Shanghai), and Sigma (America). The detected sugar components include 9 monosaccharides and 4 disaccharides (**Table 3**).

**Table 3:** Ion pair information of different sugar components.

Index	Class	Mol. Weight (Da)	Q1 (Da)	Rt (min)	Compounds	KEGG ID
Ara	Monosaccharide	150	307	4.35	D-Arabinose	C00216
Xylitol	Monosaccharide	152	307	4.723	Xylitol	C00379
Rha	Monosaccharide	164	321	4.834	L-Rhamnose	C00507
Fuc	Monosaccharide	164	117	5.051	L-Fucose	C01019
Fru	Monosaccharide	180	364	6.168	D-Fructose	C10906
Gal	Monosaccharide	180	319	6.354	D-Galactose	C00124
Glu	Monosaccharide	180	364	6.407	Glucose	C00031
Sorbitol	Monosaccharide	182	319	6.78	D-Sorbitol	C00794
Inositol	Monosaccharide	180	265	8.186	Inositol	C00137
Suc	Disaccharide	342	437	13.582	Sucrose	C00089
Lac	Disaccharide	342	361	14.208	Lactose	C00243
Mal	Disaccharide	342	361	14.822	Maltose	C00208
Tre	Disaccharide	342	361	14.9	Trehalose	C01083

### 2.5 GC-MS data evaluation

The Agilent MassHunter qualitative and quantitative software was used for data processing. The total ion current diagram is shown in **Figure S1**. It can be seen from this study that the repeatability and reliability of QC sample data are well, as shown in **Figure S2**. Attached **Table S1** is the linear equation for different carbohydrate species.

Calculation:


Sugar content(mg/g)=BCE/D/F/1000000

B: Sugar concentration value (μg/m)

C: Volume of solution for constant volume (μL)

D: Volume of supernatant (μL)

E: Volume of extract (μL)

F: Weighed sample mass (g)

### 2.6 Statistical analysis and plotting

The mean of three replicates was used to express all berry quality parameters and different sugar data. IBM SPSS v25.0 software (IBM SPSS Inc., Chicago, IL, USA) was employed to analyze Duncan’s test at a different level of significance. Pearson’s correlation coefficient was used to determine the correlation between variables. R software was utilized to conduct principal component analysis (PCA) and plot the grapes.

## 3 Results

### 3.1 Basic berry quality indexes of different grape varieties at maturity

**Figure 1:**
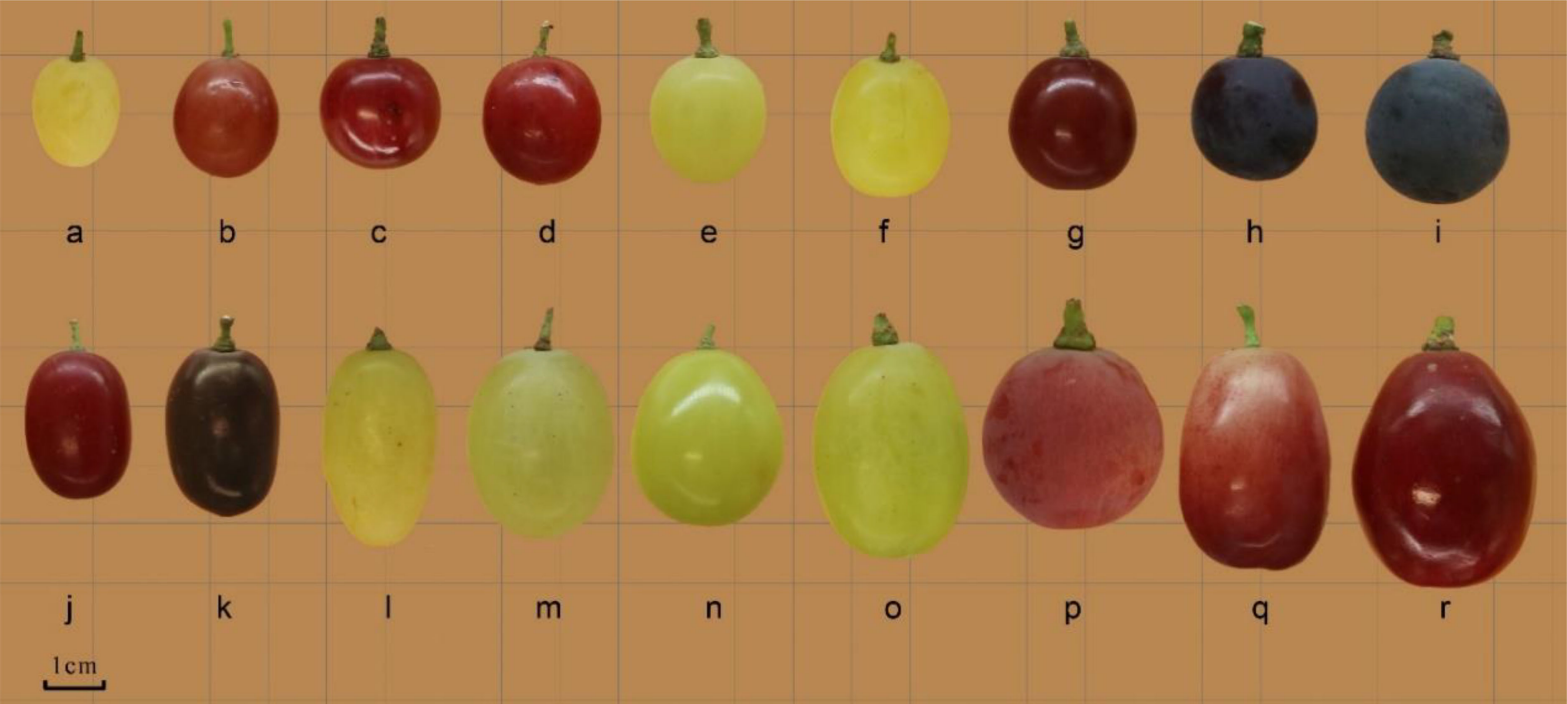
Fruit morphology of different grape cultivars. (a. Thompson Seedless; b. Bronx Seedless; c. Huozhouhongyu; d. Flame Seedless; e. Wuhecuibao; f. Melissa; g. Yatomi Rosa; h. Huozhouheiyu; i. Summer Black; j Crimson Seedless; k. Black Monukka; l. Centennial seedless; m. Munake; n. Shine-Muscat; o. Victoria; p. Red Globe; q. Rizamat; r. XinYu).

The phenotypes, such as appearance and morphology, of the 18 grape varieties are known for their own characteristics at fruit maturity (**Figure 1**). The basic characteristics of each grape variety at fruit maturity are measured, including weight, size, firmness, and total acid, and presented in **Table 4**. Based on the data in the table, it is evident that all of the varieties used in this study differ significantly. For instance, results showed that in terms of individual bunch weight, ‘XinYu’ has the largest bunch weight of 954.97g, followed by ‘Red Globe’, and so on. The ‘Wuhecuibao’ variety had the smallest panicle weight of 201.46g, while the panicle weight of ‘XinYu’ was nearly five times higher. A large variation was observed in the longitudinal diameter of individual grape bunches among different varieties. The results showed that bunch longitudinal diameter ranged from 17.58 to 30.78 cm. The highest bunch longitudinal diameter was observed in ‘Centennial seedless’ and the smallest was observed in ‘Wuhecuibao’. The bunch longitudinal diameter of ‘Centennial seedless’ was 75.08% larger than ‘Wuhecuibao’. In terms of bunch diameter, ‘Yatomi Rosa’ had the largest diameter of 22.21cm, while ‘Wuhecuibao’ had the smallest bunch diameter of 8.49 cm. The bunch diameter of ‘Yatomi Rosa’ was 161.60% larger than that of ‘Wuhecuibao’.

**Table 4:** Basic berry quality indexes of 18 varieties used in the present study.

Varieties	Panicle weight (g)	Bunch longitudinal diameter (cm)	Bunch diameter (cm)	Bunch shape index	Berry longitudinal diameter (mm)	Transverse diameter (mm)	Berry shape index	Single berry weight (g)	Soluble solids (°Brix)	Total acid (%)	TTS/TA	Harvest date
Summer Black	241.74±30.34i^jIJ^	22.27±1.58^eCD^	10.92±0.55^ijFG^	2.06±0.21^bcBCD^	19.46±0.54^efF^	18.3±0.56^fgEF^	1.06±0.01^ghG^	4.40±0.28^eE^	25.12±0.78^aA^	0.48±0.02^cdCD^	52.43±0.21^deCD^	8.11
Bronx Seedless	372.64±9.31^fghFGH^	27.54±1.60^abcdABC^	11.90±0.38^ghijFG^	2.31±0.07^abAB^	19.24±0.20^efF^	16.48±0.20^gFG^	1.17±0.01^fgEFG^	3.04±0.07^hGH^	17.25±0.22^gEF^	0.53±0.04^bcC^	33.00±1.93^ijkGH^	8.25
Crimson Seedless	600.16±37.10^cBCD^	25.10±0.24^cdeABC^	17.38±0.50^bcdeABCDE^	1.45±0.03e^fCDEF^	20.42±0.40^eF^	13.86±0.75^hGH^	1.48±0.06^cC^	2.30±0.14^iH^	22.41±0.13^bB^	0.36±0.03^eEF^	63.23±5.35^cB^	9.28
Flame Seedless	468.23±41.20d^eEF^	26.23±2.32^abcdeABC^	15.44±2.22^cdefghBCDEF^	1.73±0.09^cdefBCDEF^	17.08±0.49^fFG^	16.60±0.70^gFG^	1.03±0.04^ghG^	2.75±0.38^ghGH^	24.71±0.53^aA^	0.48±0.01^bcdCD^	51.24±1.59^deCD^	8.18
Rizamat	611.36±13.16^cBC^	30.54±1.21^abA^	20.84±1.43^abAB^	1.48±0.08^efCDEF^	35.43±0.55^aA^	20.81±0.60^deCDE^	1.71±0.05^bB^	7.84±0.52^cC^	21.89±0.49^bcBC^	0.49±0.01^bcCD^	44.68±1.64^efgDEF^	8.19
XinYu	954.97±12.30^aA^	29.58±1.37^abcAB^	19.44±0.67^abcA^B^C^	1.52±0.02^defCDEF^	29.07±0.93^cCD^	27.74±0.78^aA^	1.05±0.02^ghG^	11.59±0.28^aA^	21.93±0.18^bcBC^	0.29±0.02^fFG^	76.92±5.31^bA^	9.13
Wuhecuibao	201.46±13.39^jJ^	17.58±0.65^fD^	8.49±0.56^jG^	2.10±0.22^bcABC^	18.20±0.93^efF^	17.85±0.82^fgEF^	1.03±0.09^ghG^	3.37±0.15^fgFG^	19.96±0.23^deCD^	0.23±0.01^fG^	86.24±2.23^aA^	8.16
Shine Muscat	607.58±39.75^cBC^	29.13±1.33^abcAB^	16.05±2.79^cdefBCDEF^	1.96±0.43^bcdeBCDE^	25.40±0.61^dE^	25.24±0.27^bAB^	1.01±0.03^hG^	7.55±0.13^cC^	21.98±0.84^bcBC^	0.46±0.01^cdCD^	48.23±1.56^defCDE^	9.12
Victoria	509.01±34.24^dCDE^	23.43±1.09^deBCD^	15.86±0.63^cdefgBCDEF^	1.48±0.05^efCDEF^	33.06±0.20^bAB^	22.43±0.31^cdBCD^	1.47±0.03^cC^	10.12±0.21^bB^	14.93±0.21^hG^	0.27±0.02^fG^	56.19±3.47^cdBC^	8.17
Black Monukka	419.13±20.10^efgEFG^	27.88±1.24^abcdABC^	13.63±0.64^efghiDEFG^	2.06±0.17^bcBCD^	25.33±0.73^dE^	17.97±0.50^fgEF^	1.41±0.02^cdCD^	5.52±0.32^dD^	18.73±0.11^efDE^	0.67±0.01^aA^	28.06±0.29^kH^	8.28
Red Globe	709.47±14.85^bB^	28.25±2.34^abcABC^	17.77±1.46^bcdABCDE^	1.60±0.12^cdefCDEF^	26.60±0.53^dDE^	25.05±1.40^bAB^	1.07±0.09^ghG^	7.97±0.25^cC^	20.02±0.12^deCD^	0.55±0.04^bBC^	36.77±2.43^ghijFGH^	9.16
Thompson Seedless	332.76±16.53^ghGHI^	27.75±0.84^abcdABC^	11.65±0.31^hijFG^	2.38±0.05^abAB^	14.28±0.37^gG^	10.90±0.11^iI^	1.31±0.02^deCDE^	2.29±0.08^hH^	24.65±0.22^bcBC^	0.64±0.01^aA^	33.97±0.65^hijkFGH^	8.21
Centennial Seedless	277.48±14.10h^ijHIJ^	30.78±0.99^aA^	11.26±0.46^ijFG^	2.74±0.08^aA^	26.55±0.49^dDE^	12.81±0.28^hiHI^	2.07±0.05^aA^	4.09±0.28^efEF^	19.03±0.56^efDE^	0.67±0.02^aA^	28.49±1.54^jkH^	9.06
Munake	336.51±23.28^ghGHI^	24.74±1.21^cdeABC^	12.56±1.26^fghiEFG^	2.02±0.26^bcdBCDE^	26.43±1.40^dDE^	24.94±0.96^bAB^	1.06±0.03^ghG^	8.29±0.08^cC^	17.71±0.56^fgEF^	0.49±0.01^bcCD^	36.08±1.24^hijkFGH^	9.27
Yatomi Rosa	702.41±25.31^bB^	29.14±2.37^abcAB^	22.21±1.43^aA^	1.33±0.14^fEF^	31.20±0.82^bcBC^	23.15±0.88^bcBC^	1.35±0.03^cdeCDE^	8.21±0.21^cC^	16.41±0.15^gFG^	0.42±0.01^deDE^	39.4167±1.02^ghiEFGH^	8.22
Huozhouheiyu	431.11±33.24^defEFG^	22.58±0.66^eCD^	16.39±0.08^cdefBCDEF^	1.38±0.04^fDEF^	17.98±0.70^efF^	18.09±0.85^fgEF^	1.00±0.03^hG^	5.68±0.13^dD^	20.59±0.61^cdBCD^	0.49±0.01^bcCD^	42.08±2.32^fghDEFG^	8.23
Huozhouhongyu	296.85±23.41g^hiHIJ^	17.71±0.46^fD^	14.24±0.61^defghiCDEF^	1.25±0.02^fF^	20.05±0.99^eF^	18.39±0.50^fgEF^	1.09±0.05^ghFG^	2.74±0.07^ghGH^	20.72±0.44^cdBCD^	0.61±0.05^aAB^	34.33±3.29^hijkFGH^	8.26
Melissa	495.35±42.44^deDE^	25.81±1.82^bcdeABC^	18.22±1.99^bcdABCD^	1.43±0.10^fCDEF^	25.14±1.89^dE^	19.87±0.84^efDE^	1.26±0.06^efDEF^	4.67±0.38^eDE^	21.28±0.80^bcdBC^	0.48±0.02^cdCD^	44.91±3.49^efgDEF^	9.12

The different lowercase letters in the column indicate significant differences between cultivars (p < 0.05), different capital letters indicate significant differences between cultivars (p < 0.01).

Grape berries of different varieties with different colors and shapes are shown in **Figure 1**. The parameters related to the berry index were also measured to understand the structural variation among the varieties. The longitudinal diameter of barriers ranged from 14.28 to 35.43mm. The longitudinal diameter of ‘Rizamat’ was the largest, and ‘Thompson seedless’ had the smallest longitudinal diameter. The fruit longitudinal diameter of ‘Rizamat’ was 148.11% larger than that of ‘Thompson seedless’. In terms of berry transverse diameter, ‘XinYu’ was in top rank with 27.74 mm, and ‘Thompson seedless’ is the smallest, reaching 10.9 mm. The berry transverse diameter of ‘XinYu’ was 53.34% larger than that of the smallest variety. As for single berry weight, ‘XinYu’ was the largest, and ‘Thompson seedless’ was the smallest.

Total soluble solid (TSS) is among the most essential attributes that determine the quality of fruits. TSS is dominated by total sugar content, with a minor contribution from soluble proteins, amino acids, and other organic materials (Xu et al., 2022). In this study, the TSS of ‘Summer Black’ and ‘Flame Seedless’ was found to be 25.12 and 24.71° Brix, respectively, which was higher than other grape varieties, while ‘Victoria’ had the lowest TSS of 14.93° Brix. ‘Summer Black’ and ‘Flame Seedless’ had 68.25% and 65.50% higher TSS than ‘Victoria,’ respectively. Furthermore, ‘Black Monukka’ and ‘Centennial Seedless’ had the highest total acid content of 0.67%, while ‘Victoria’ had the lowest total acid content of 0.27% among all grape varieties studied.

The study conducted by Huang et al. (2021) used the soluble solids to acid ratio as an indicator to evaluate grape maturity and flavor. The TSS/TA ratio was calculated for all grape varieties, and the results showed that the ratio ranged from 86.24 (Wuhecuibao) to 28.06 (Black Monukka), indicating significant differences among the varieties. Specifically, ‘Wuhecuibao’ had a TSS/TA ratio that was 207.34% higher than that of ‘Black Monukka’. Berry maturity is a crucial stage for harvesting, and fruit quality often depends on maturity indices (Niimi et al., 2017; Niimi et al., 2018). The grape varieties used in this study were classified as early and late based on their harvest dates. ‘Summer Black’, ‘Victoria’, and ‘Flame seedless’ matured early, while ‘Crimson seedless’ and ‘Munake’ matured relatively late.

### 3.2 Variation in monosaccharide and disaccharide substances amongst grape varieties

**Figure 2:**
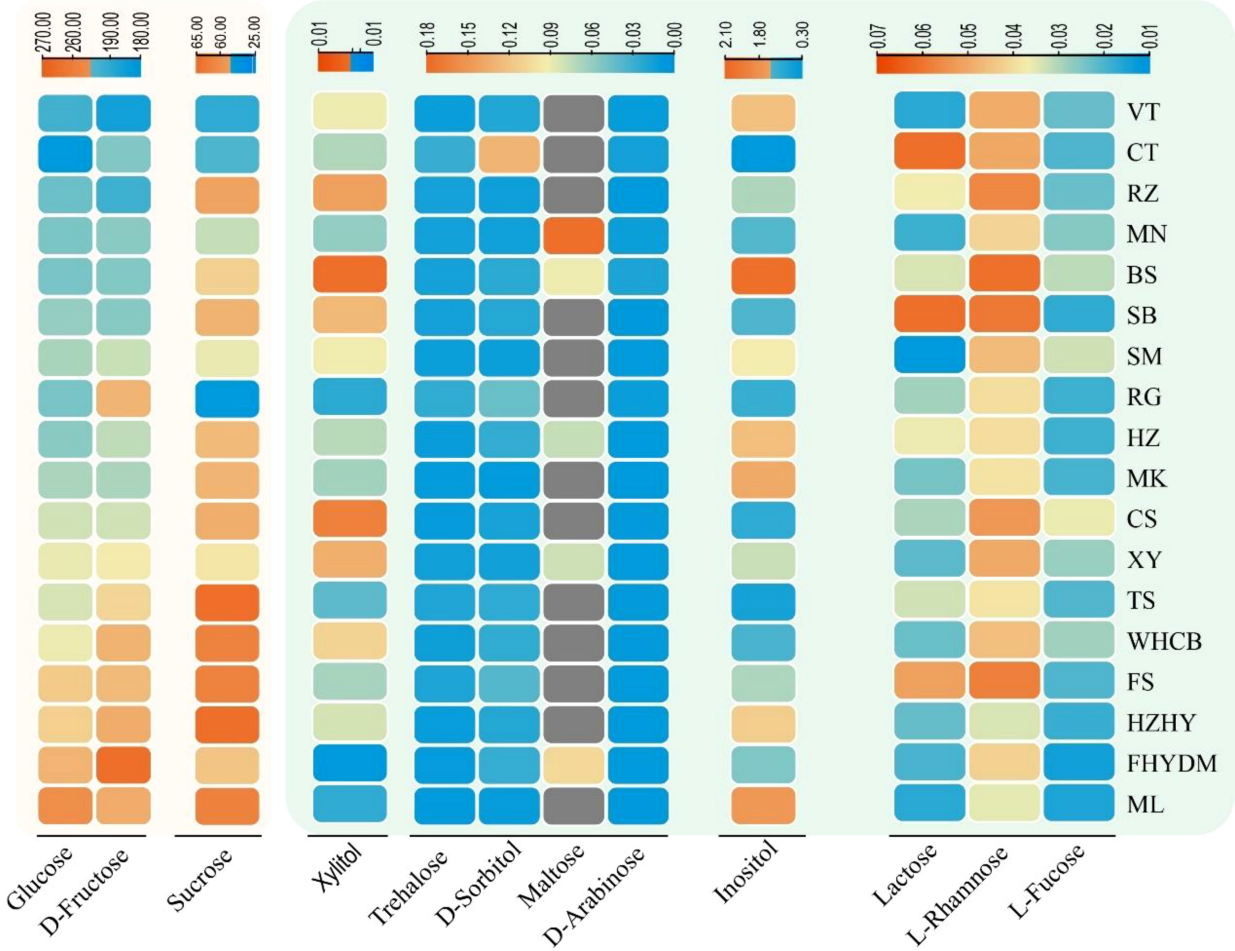
Variation and distribution of sugar contents in 18 grape varieties are presented in form of heatmap. The sugar content was measured in mg/g. Thompson Seedless (TS); Bronx Seedless (BS); Huozhouhongyu (HZHY); Flame Seedless (FS); Wuhecuibao (WHCB); Melissa (ML); Yatomi Rosa (FHYDM); Huozhouheiyu (HZ); Summer Black (SB); Crimson Seedless (CS); Black Monukka (MK); Centennial Seedless (CT); Munake (MN); Shine-Muscat (SM); Victoria (VT); Red Globe (RG); Rizamat (RZ); XinYu (XY).

The GC-MS analysis revealed significant differences in the amounts of sugar components present in the berries of the 18 grape varieties tested (**Figure 2**). The heatmap showed that, with the exception of maltose, all identified sugar components were detected in all tested varieties, whereas maltose was only detected in five varieties. Notably, the concentration of the other nine sugar components was found to be low, while D-fructose and glucose were present in high concentrations, followed by sucrose. The heatmap depicts the average concentration of different sugar components among the varieties.

#### 3.2.1 Variation in content of D-fructose among grape varieties

**Figure 3:**
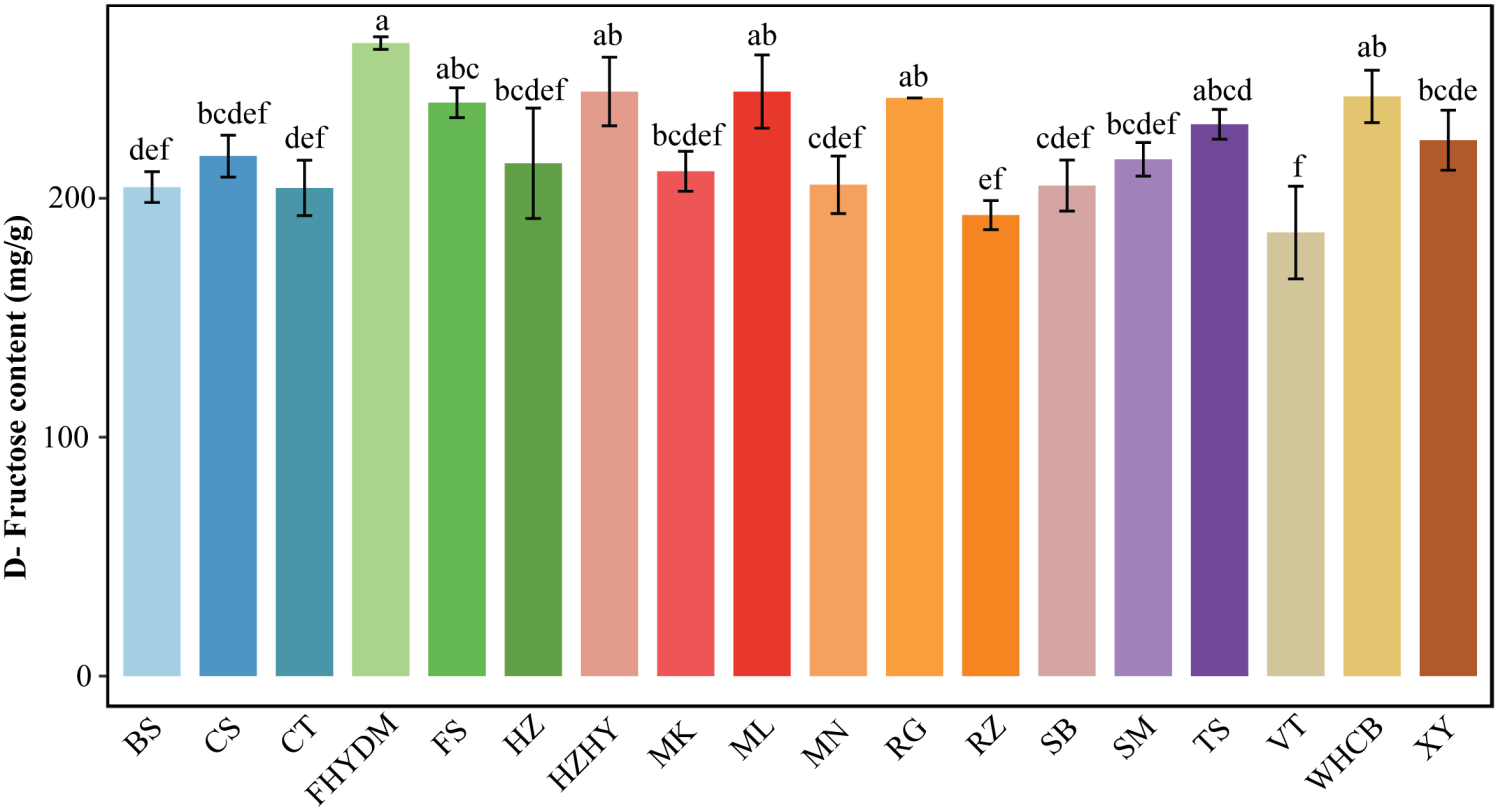
Analysis of D-fructose content in 18 varieties Bars of different colors represent the grape varieties used in the current study. The D-fructose content was measured in mg/g. The alphabets on the error bars show statistically significant values between varieties. Values are the means of a minimum of three replicates and expressed as means ± standard deviation (SD). Different superscripts in the same row indicate statistical differences using the Duncan test.

Fructose is a crucial carbohydrate in nutrition, and though it is a monosaccharide like glucose and galactose, it has a distinct flavor. Upon analyzing various sugar components, it was found that D-fructose content was highest in all varieties, constituting between 42.68% to 50.95% of the total sugar in different types. D-fructose levels in varieties ranged from 185.66 to 265 mg/g, with the top three varieties containing the highest D-fructose contents being ‘Yatomi Rosa’, ‘Melissa,’ and ‘Wuhecuibao.’ Of these, ‘Yatomi Rosa’ had the highest D-fructose content at 265 mg/g, followed by ‘Wuhecuibao,’ Red Globe, and ‘Flame Seedless’ (**Figure 3**). The variety with the lowest D-fructose content is ‘Victoria,’ containing only 185 mg/g. The difference in D-fructose content between ‘Yatomi Rosa’ and ‘Victoria’ is the largest, with ‘Yatomi Rosa’ having 43.24% more D-fructose content than ‘Victoria.’ Additionally, there is a significant difference in D-fructose content between ‘Flame Seedless’ and ‘Victoria’ grapes, with ‘Flame Seedless’ having 29.72% higher D-fructose content than ‘Victoria’ grapes (**Figure 3**).

#### 3.2.2 Uneven distribution of glucose content in grape varieties

**Figure 4:**
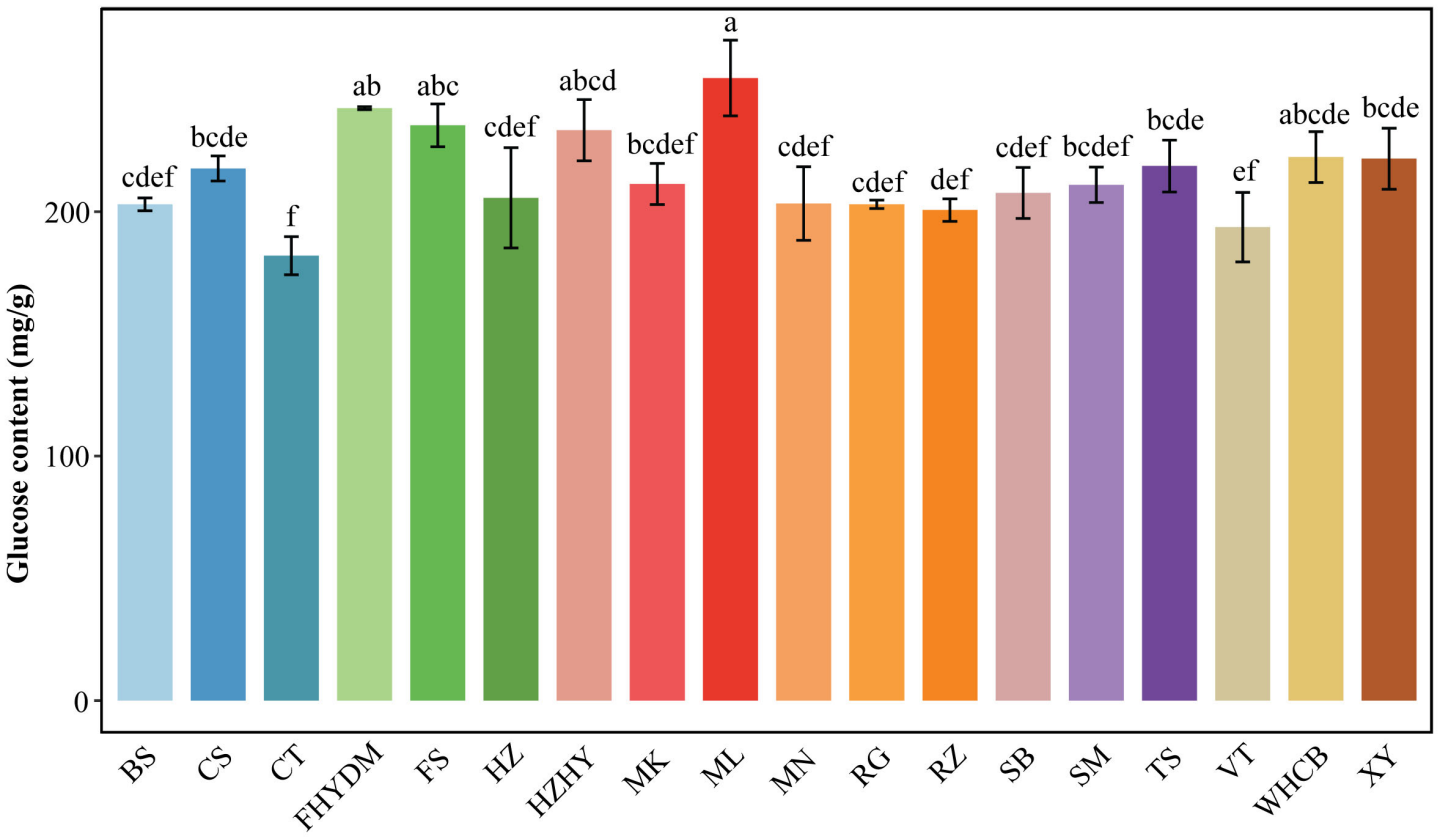
Analysis of glucose content in 18 varieties. Bars of different colors represent the grape varieties used in the current study. The D-fructose content was measured in mg/g. The alphabets on the error bars show statistically significant values between varieties. Values are means of minimum three replicates and expressed as means ± standard deviation (SD). Different superscripts with the same row indicate statistical differences using the Duncan test at different level of significance.

The glucose range in total sugar across the 18 grape varietals varied from 42.13% to 46.80%. The top three varieties with the highest glucose levels are ‘Melissa’, ‘Yatomi Rosa’, and ‘Flame Seedless’, with glucose contents of 254 mg/g, 242 mg/g, and 235 mg/g, respectively (**Figure 4**). ‘Centennial Seedless’ and ‘Victoria’ have the lowest glucose content among all varieties, with ‘Centennial Seedless’ having the lowest glucose level of only 182 mg/g, followed by ‘Victoria’ with 193 mg/g. ‘Flame Seedless’ has a glucose level that is 21.76% higher than ‘Victoria’, and the glucose content of ‘Melissa’ is 40% higher than that of ‘Centennial Seedless’. The highest difference in glucose content is between ‘Melissa’ and ‘Centennial seedless’. The glucose content in the majority of varieties ranged from 193.6 to 254.6 mg/g (**Figure 4**). Moreover, results showed that glucose content in varieties did not relate to maturity duration. Some varieties with late and early maturity had similar glucose levels. For instance, there was no significant difference in glucose levels between ‘Summer Black’ and ‘Munake’, which are early and late maturity varieties.

#### 3.2.3 Variation in sucrose content in different varieties

**Figure 5:**
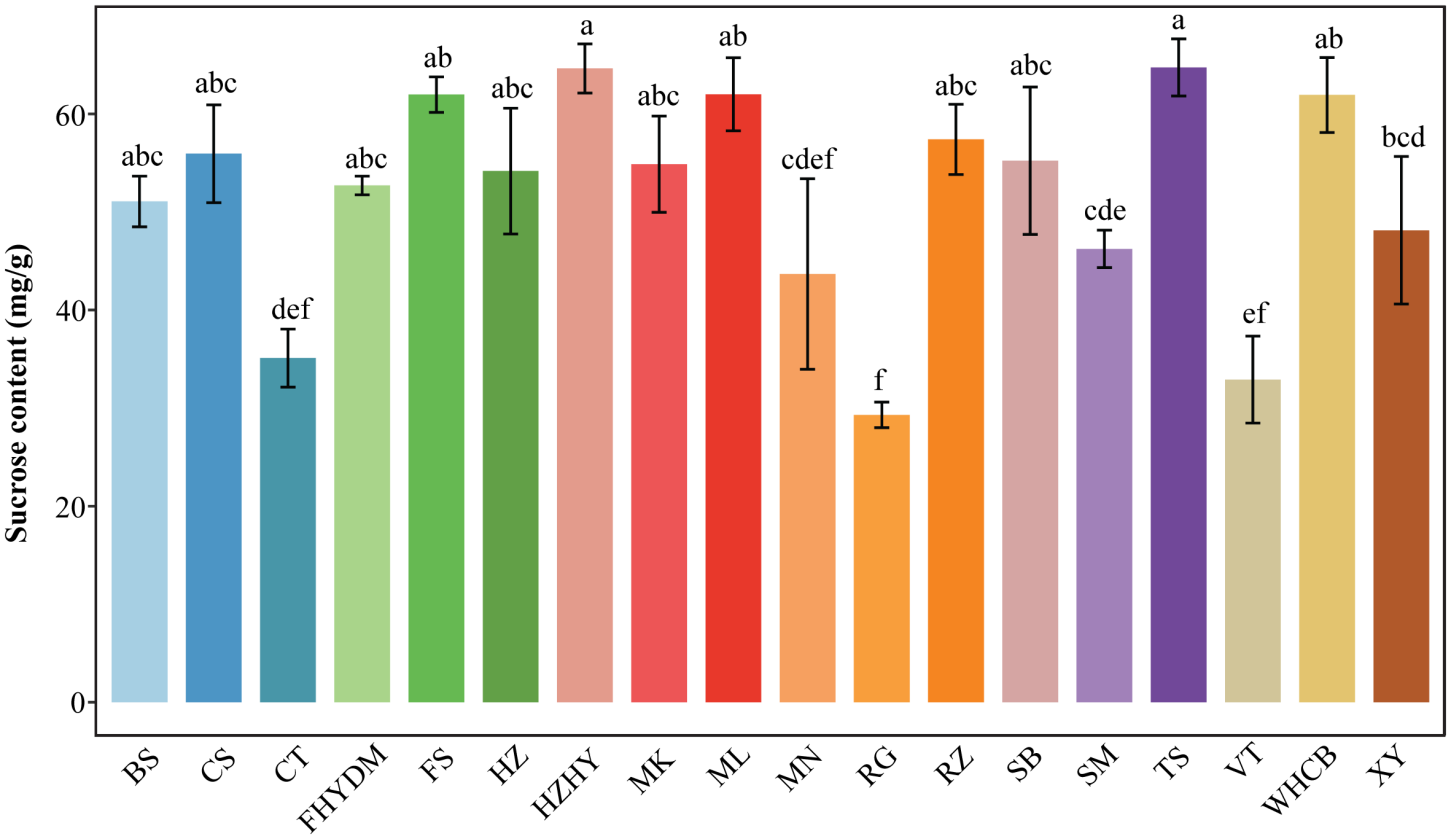
Variation in sucrose content across 18 varieties. Values are means of minimum three replicates and expressed as means ± standard deviation (SD). Different superscripts with the same row indicate statistical differences using the Duncan test.

Sucrose is the third most common type of sugar found in grape berries, following D-fructose and glucose. The amount of sucrose present in the total sugar content of the 18 grape varieties tested ranged from 6.17% to 12.69%. The three grape varieties with the highest sucrose content were ‘Thompson Seedless’, ‘Huozhouhongyu’, and ‘Melissa’, with ‘Thompson Seedless’ having the highest sucrose content at 64 mg/g (**Figure 5**). The three varieties with the lowest sucrose content were ‘Red Globe’, ‘Victoria’, and ‘Centennial Seedless’, with ‘Red Globe’ having the lowest sucrose content at 29 mg/g. The difference in sucrose content between the lowest (Melissa) and highest (Thompson Seedless) was 121%.

#### 3.2.4 Minor and trace sugar components

**Figure 6:**
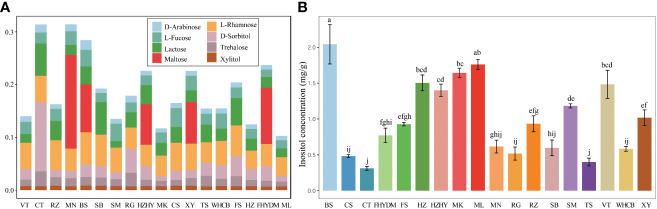
Analysis of nine trace sugar content in18 grape varieties. **(A)** The stacked bar plot shows share of eight different trace sugar in different varieties. **(B)** The content of inositol in grape varieties. Colour bars shows different varieties. The significant differences are shown with the letter on top of error bar.

The estimated sugar content in grape berries, as determined by GC-MS analysis, shows that there are some sugar units present in small quantities. D-fructose, glucose, and sucrose are present in higher concentrations, while some monosaccharides and disaccharides, including D-arabinose, inositol, lactose, maltose, trehalose, and xylitol, are detected at very low concentrations. Among these, the content of inositol was higher than that of other trace sugar components, with concentrations in grape varieties varying from 0.31 to 2.04 mg/g. Notably, the content of maltose was detected in only a few varieties, including ‘Munake’, ‘Bronx seedless’, ‘Huozhouhongyu’ and ‘Yatomi Rosa’, with concentrations ranging from 0.01 to 0.07 mg/g. The details about the concentration of trace sugar components are displayed in **Figure 6A**, where significant differences can be clearly seen in the concentration of inositol in different varieties. The results revealed that the concentration of inositol was exceptionally higher in ‘Bronx seedless’ (**Figure 6B**).

### 3.3 Differences and comparative analysis of total sugar in fruit of different genotype grape varieties

**Figure 7:**
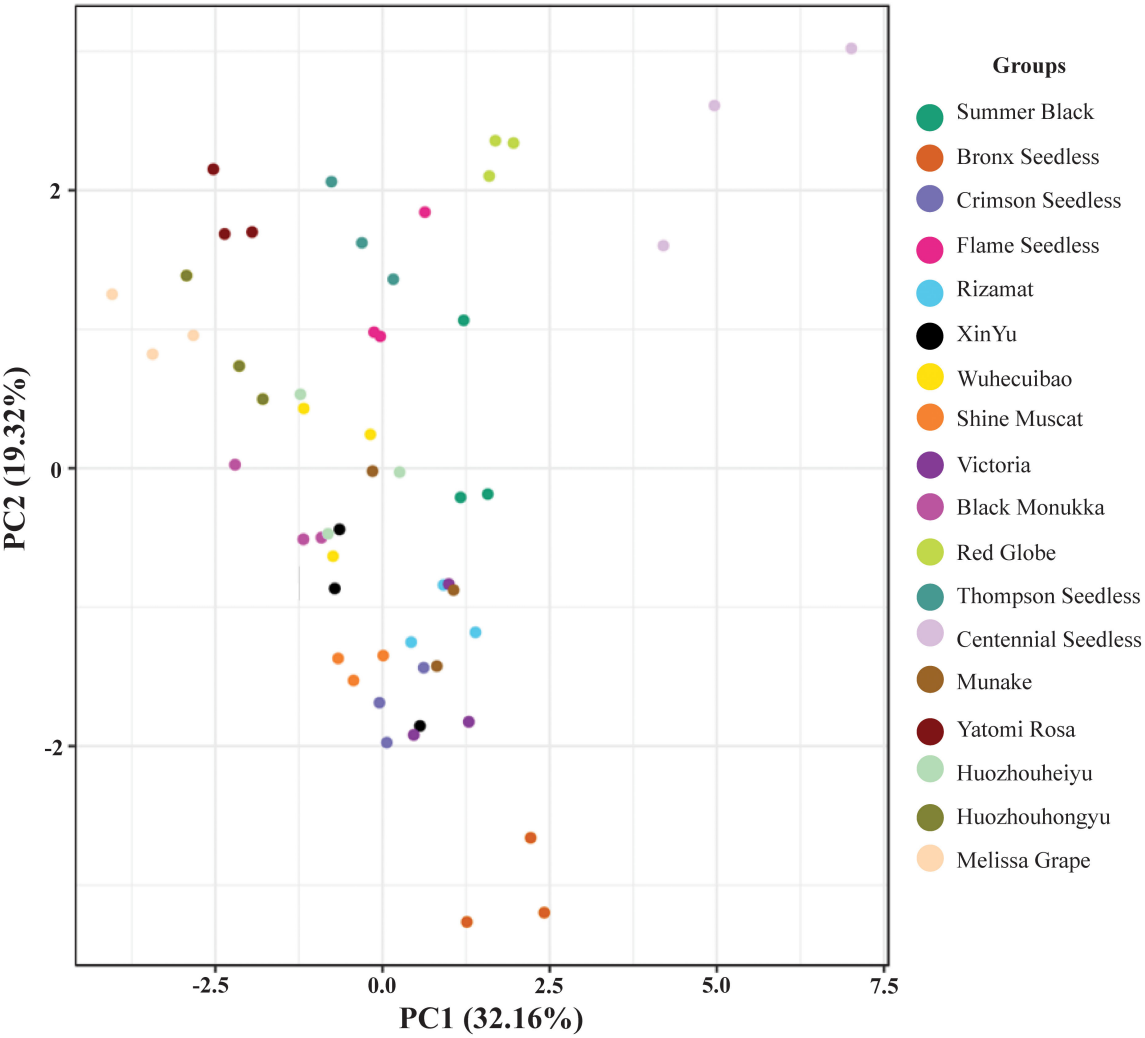
Principal component analysis of sugar content in 18 cultivars. Different color dots represents the various varieties of grapes. Multiple dots of same color shows replication of samples.

Based on the analysis of the components and contents of various sugars in different grape varieties using GC-MS, principal component analysis (PCA) was conducted to investigate the differences both within and among replicates. The results, depicted in **Figure 7**, reveal that the three replicates of each of the 18 grape varieties were closely clustered together, suggesting good repeatability and ensuring the accuracy of the data. Moreover, the samples of different grape varieties were clearly distinguishable, indicating that the sugar composition and content among different grape varieties differed significantly, reaching a significant or extremely significant level. The findings highlighted the substantial variation in sugar composition and content among grape varieties.

**Figure 8:**
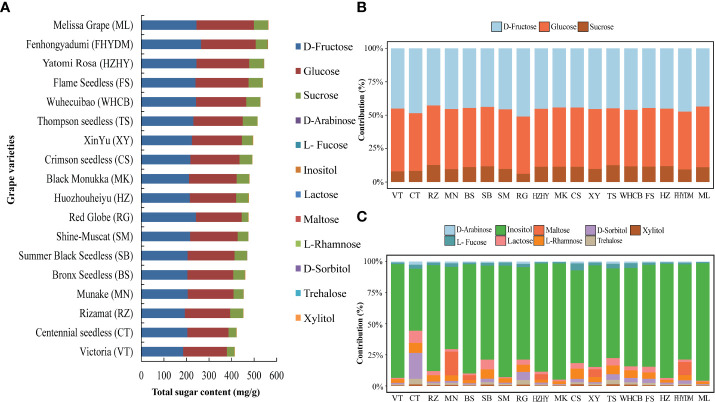
Analysis of total sugar content in 18 cultivars and percentage share of major sugar and trace sugar in each variety. **(A)** Total sugar content in all grape varieties **(B)** The percentage contribution of major sugar components to all grape varieties. **(C)** The percentage share of trace sugar components in different varieties is shown with different color bars.

According to **Figure 8A**, the total sugar content in these grape varieties is determined by the sum of monosaccharides and disaccharides detected by GC-MS, including their absolute values. ‘Melissa’ has the highest total sugar content, followed by ‘Yatomi Rosa’ and ‘Huozhouhongyu’, and then ‘Flame seedless’, ‘Wuhecuibao’, ‘Thompson seedless’, and ‘Xin Yu’, among others. Conversely, ‘Victoria’, ‘Centennial Seedless’, and ‘Rizamat’ were found to have lower sugar content. In 18 varieties, D-fructose, glucose, and sucrose contribute to over 99% of the total sugar content, with D-fructose slightly higher than glucose in 12 of them (**Figure 8B**). Additionally, inositol was identified as a higher contributor to the minor sugar components in all grape varieties (**Figure 8C**).

### 3.4 Comparison and analysis of sugar quality of different grape varieties

**Figure 9:**
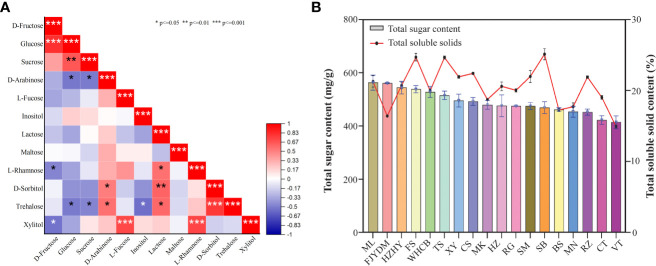
Correlation analysis of total sugar content and soluble solids. **(A)** Spearman correlation coefficients among different sugar contents. **(B)**The total sugar content is shown on left y-axis and right y-axis shows percentage of total soluble solids. * p<=0.05 ** p<=0.01 *** p<=0.001.

The correlations between various sugar components are presented in **Figure 9A** using Spearman correlation coefficients. The correlation matrix revealed that some sugar components showed a positive correlation with others. For instance, Xylitol content exhibited a positive correlation with L-fucose (p<0.001) and L-rhamnose (p<0.001). Similarly, lactose content was positively correlated with D-sorbitol (p<0.01), L-rhamnose (p<0.05), and trehalose (p<0.05) content. A strong correlation between sucrose content and glucose content was observed. Furthermore, glucose content in varieties was found to have a strong correlation with D-fructose content (p<0.001). Additional positive and negative correlations were identified, and further details are presented in **Figure 9A**.

Results shown in **Figure 9B** revealed that there is no significant positive correlation between total sugar and soluble solid content. For example, although ‘Yatomi Rosa’ has the second-highest total sugar content, its proportion of soluble solids is lower. Similarly, ‘Summer Black’ has the highest soluble solids, but its total sugar content is not particularly high. In contrast, ‘Flame Seedless’ has relatively high levels of both total sugar and soluble solids, while ‘Victoria’ has lower levels of both. There are significant differences in sugar and total soluble content among some varieties. For instance, the total soluble solid content in ‘Summer Black’, ‘Flame Seedless’, and ‘Thompson Seedless’ is similar, but the total sugar content varies greatly. Therefore, the plot showing the relationship between total sugar and total soluble solids indicates that there is no positive correlation between these parameters.

## 4 Discussion

### 4.1 Variation in basic berry characteristics

The morphology and characteristics of berries vary among the varieties and different species of grapes (**Table 4**). Quantifying the phenotypic parameters of grape berries and bunches is important for precision agriculture (Liu et al., 2022a). The cultivated grapes are known to have high variation compared with wild resources, which largely resemble round berries in shape (Rodríguez et al., 2011; Zhang et al., 2021). In many fruit crop markets, demand is closely associated with the shape and quality of fruit crops. Varietal development programs are also associated with fruit shape in many fruit crops (van der Knaap et al., 2014; Zhang et al., 2021). The morphological characteristics observed in our study showed that the popular grape varieties grown in the Xinjiang region are round to oval in shape. Many studies have shown that berry appearance is highly associated with berry index and appears to influence the acceptability and preferences of consumers when it comes to fresh fruit consumption (Lecourieux et al., 2014; Zhang et al., 2022b). The studies by Zhang et al. (2022a) and Zhang et al. (2022b) revealed that in some regions, spine grapes are eaten because of their large shape and flavor. The berries are characterized by high variability in grapes and these traits are also used to describe different grape varieties and domestication processes (Barbagallo et al., 2020). We measured various berry traits to highlight the phenotypic diversity of berries in different varieties grown in Xinjiang. The varieties used in the current investigation showed higher variability in various berry characteristics. The significant differences were reported in bunch parameters, berry parameters, berry index, TSS, total acid and harvesting time. The technological maturity parameters of Italian table grapes were measured in some popular varieties. The shorting of varieties on a commercial level showed significant variation in different chemical parameters, including TSS, TA, TSS/TA, pH, and different major sugar components (Segade et al., 2013). Barbagallo et al. (2020) used a number of grape varieties from the Sicilian genetic pool and precisely observed the variability in seed and berry traits. Similar research was carried out by Bouby et al. (2013) to identify the difference in elongation of the pip body between primitive cultivars and highly domesticated cultivars. The results revealed that traits of domestication are related to the strength of selection pressure (Bouby et al., 2013).

### 4.2 Distribution of sugar contents in grape varieties

The quality of grape berries is mainly determined by the type and amount of sugar, soluble solids, and organic acids content. Among these factors, sugar content is the most important. Xinjiang is home to a wide variety of grape germplasm, but different grape varieties have varying levels of sugar. In a study of 18 grape varieties, we found that fruit size, shape, and color characteristics differed at maturity, as did sugar content. Sugar accumulation during fruit development has been extensively studied in various species, and the amount of total soluble sugars typically increases with growth, reaching a peak at maturity or ripening (Basson et al., 2010; Li et al., 2012; Cao et al., 2013). However, the patterns and concentrations of sugar accumulation can differ between species. Glucose and fructose are typically the major proportion of soluble sugars in most fruits, while sucrose is predominant in some species like peaches, citrus, and litchi (Yakushiji et al., 1996; Desnoues et al., 2014; Li et al., 2020).

In our studies, we observed a similar pattern of sugar distribution, where glucose and fructose contributed the majority of sugars. The sugar-to-acid ratio reflects fruit taste, and the flavor of grape berries is closely related to sugar-acid content (Guo et al., 2017; Zhang et al., 2022b). Fructose and glucose are the most common sugars in most fruits, and they also contribute to the flavor of grape berries. Other metabolic changes occur during grape berry ripening, such as the accumulation of sugars in the form of glucose and fructose in the berry vacuoles (flesh and skin) following sucrose translocation from the leaves (Durán-Soria et al., 2020). Recent research by Li et al. (2020) compared sugar profiles in major fruit crops and found that glucose and fructose were the most abundant sugars, which is consistent with our findings. However, sucrose was found to be the predominant sugar in some fruits, including peaches.

The recent studies conducted on grapes have shown that the sugar content of wild grapes is primarily divided into fructose and glucose, as reported by Liu et al. (2006); Jiang et al. (2017); Zhang et al. (2022b) and Segade et al. (2013). Our current study supports these findings and indicates that the sugar composition and level vary significantly across grape varieties, as observed in previous studies on grapes (Zhang et al., 2022b) and citrus (Zhou et al., 2018). Specifically, our analysis of individual sugar components revealed that fructose, glucose, and sucrose are the three primary sugar components in grapes, accounting for over 99% of the total sugar content. Among the 18 grape varieties studied, fructose accounted for 42.68%-50.95% of the total sugar, while glucose accounted for 42.13%-46.80%, both accumulating at significantly higher levels than sucrose and other sugar substances, consistent with the findings of Li et al. (2020) and Liu et al. (2006). Similar results were observed in the sugar concentration of other fruits, where D-fructose, sucrose, and glucose were the major sugar contributors (Ma et al., 2017). The proportion of sucrose content in total sugar ranged from 6.17% to 12.69%. Orak (2009) reported a variation in glucose content of 5.98% to 12.21% and fructose content of 5.93% to 12.66% in 24 significant grape varieties, which is similar to our findings. The carbohydrate composition on grape varieties identified by thin layer chromatography, and spectrophotometric Dubois method also revealed that glucose and fructose as major constituents in black grape varieties (Iosub et al., 2013). However, the variation in fructose and glucose ranges may be due to different analytical methods used for sugar content estimation. Interestingly, our current research found that the majority of grape varieties had trace amounts of monosaccharides and disaccharides, unlike many other studies. The concentration of lactose and maltose was measured in some Korean fruits and vegetables, but they were not present in most of them. Shanmugavelan et al. (2013) found that the concentration of trace sugar was not enough to be detected by HPLC in Campbell early and green varieties of grapes. In contrast, GC-MS-based sugar quantification in our study showed that many trace sugars were present in most of the grape varieties, with only five varieties showing the presence of maltose. The differences in the identification of various sugars can be attributed to analytical methods or variety differences. In our study, the sugar composition determined by GC-MS was combined with the data of soluble solids, and it was found that the sugar content, an important quality factor, differed significantly among the 18 grape varieties. GC-MS has been found to be effective in previous studies for the quantitation of carbohydrate intermediates (Milkovska-Stamenova et al., 2015). In short, current study allowed to characterize economically important grape varieties grown in Xinjiang grapes according to physiochemical and sugar composition.

## 5 Conclusion

Thirteen carbohydrate components were correctly detected in the berries of 18 different grape varieties using GC-MS technology. Fructose and glucose were the predominant sugar types in grape berries, followed by sucrose. However, the average content of D-arabinose, lactose, maltose, trehalose, and the other nine sugars was very low, ranging from 0.01 to 1.04 mg/g. The grape varieties with higher fructose content were ‘Yatomi Rosa’, ‘Huozhouhongyu’, and ‘Melissa’, while those with higher glucose content were ‘Melissa’, ‘Yatomi Rosa’, and ‘Flame Seedless’. ‘Thompson seedless’, ‘Huozhouhongyu’, and ‘Melissa’ were the grape varieties with a higher sucrose content. The top three varieties with higher total sugar content were ‘Melissa’, ‘Yatomi Rosa’, and ‘Huozhouhongyu’. Further analysis of total sugar and soluble solids showed no significant correlation between them. Positive and negative correlations were observed between some major and trace sugars. ‘Flame Seedless’ scored rather well on the indices of total sugar and soluble solids. ‘Flame Seedless’ and ‘Victoria’, having the same population source and maturity stage, had significant differences in sugar content and could be chosen as representatives of high- and low-sugar-type varieties for further study. Collectively, the findings suggested that a phenotypic characteristic with sugar content and type analysis can be used as a comprehensive and objective evaluation system for determining the quality of grape varieties.

## Data availability statement

The original contributions presented in the study are included in the article/**Supplementary Material**. Further inquiries can be directed to the corresponding authors.

## Author contributions

Author Contributions: Conceptualization, HZ and FZ; Data curation, HZ, FZ, VY, ZW, MP, and XW. Formal analysis, HZ, FZ, VY, ZW, SH, and MW; Funding acquisition, HZ and FZ, Methodology, HZ and FZ. Writing – review and editing, HZ, FZ, VY, DY, and CZ. All authors contributed to the article and approved the submitted version.
