# Supplementary material for: Comprehensive metabolomics-based analysis of sugar composition and content in berries of 18 grape varieties
Source: Front Plant Sci. 2023 Jun 9;14:1200071. doi: 10.3389/fpls.2023.1200071 (PMC10288860; doi:10.3389/fpls.2023.1200071)
Supplement: Supplementary file 1 [file DataSheet_1.docx]

Appendix


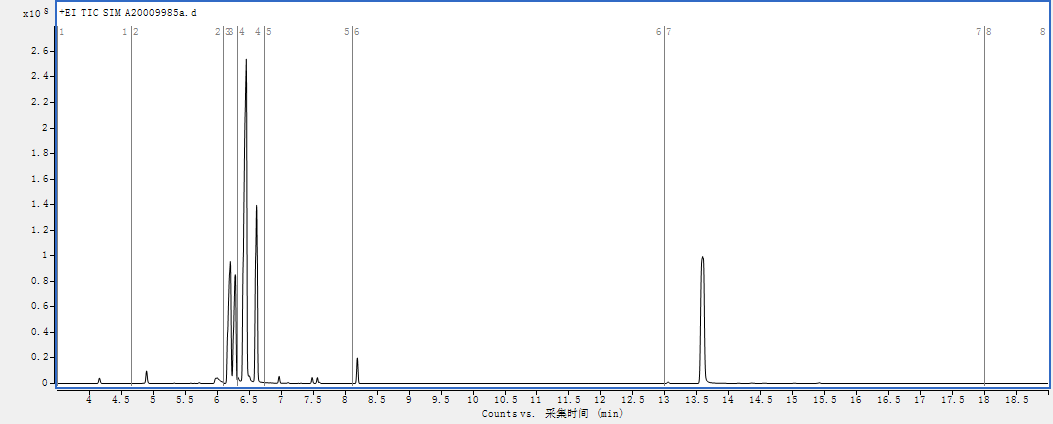


Fig. S1 Total ion flow diagram of targeted metabolome


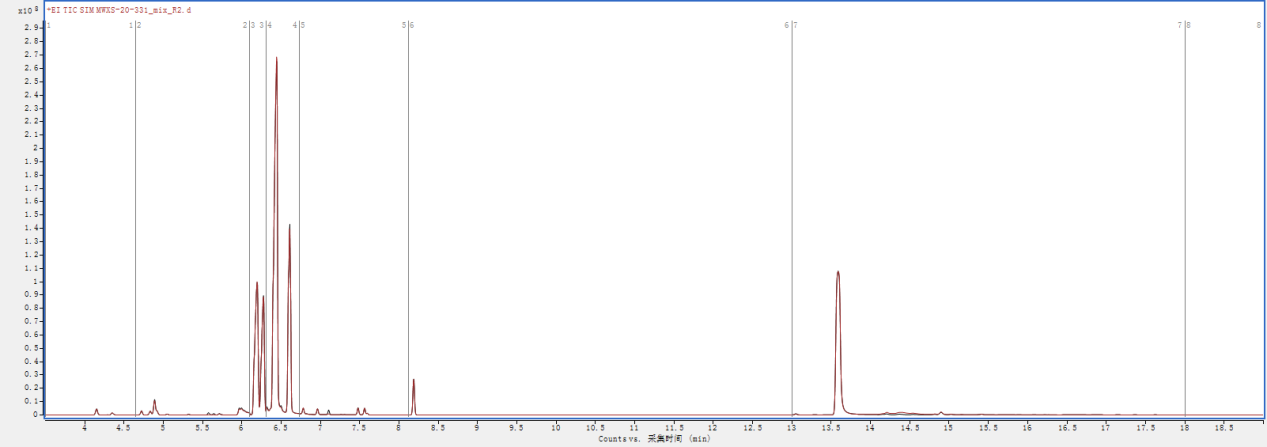


Fig. S2 The TIC overlap pattern was detected by qC-like essence spectrum of targeted metabolome

Table S1 Standard curve linear equations and correlation coefficients of saccharides

| Component | Equation | coefficient of correlation | Weight | LLOQ  (μg/ml) | ULOQ  (μg/ml) |
| --- | --- | --- | --- | --- | --- |
| D-Arabinose | y = 1.200000 x- 0.006038 | 0.997786988 | 1/x | 0.003 | 4.5 |
| Xylitol | y = 1.043136 x- 0.002090 | 0.997213518 | 1/x | 0.003 | 4.5 |
| L-Rhamnose | y = 0.134480 x- 6.734563E-004 | 0.998640648 | 1/x | 0.003 | 4.5 |
| L-Fucose | y = 1.256083x- 0.005824 | 0.998964128 | 1/x | 0.003 | 4.5 |
| D-Fructose | y = 0.050083x + 0.623718 | 0.990457509 | 1/x | 3 | 50 |
| Glucose | y = 0.015492 x + 0.203511 | 0.993489181 | 1/x | 3 | 50 |
| D-Sorbitol | y = 2.720445 x - 0.006187 | 0.998602665 | 1/x | 0.003 | 4.5 |
| Inositol | y = 0.916749 x - 0.002230 | 0.996477202 | 1/x | 0.003 | 4.5 |
| Sucrose | y = 0.261313 x - 0.004779 | 0.992244889 | 1/x | 0.03 | 50 |
| Lactose | y = 0.339665x - 0.001011 | 0.999277926 | 1/x | 0.003 | 4.5 |
| Maltose | y = 0.314412 x - 0.020187 | 0.999274159 | 1/x | 0.04 | 6 |
| Trehalose | y = 2.918488x - 0.013322 | 0.998757585 | 1/x | 0.003 | 3 |
